# The Alternative Epac/cAMP Pathway and the MAPK Pathway Mediate hCG Induction of Leptin in Placental Cells

**DOI:** 10.1371/journal.pone.0046216

**Published:** 2012-10-02

**Authors:** Julieta Lorena Maymó, Antonio Pérez Pérez, Bernardo Maskin, José Luis Dueñas, Juan Carlos Calvo, Víctor Sánchez Margalet, Cecilia Laura Varone

**Affiliations:** 1 Departamento de Química Biológica, Facultad de Ciencias Exactas y Naturales, Universidad de Buenos Aires, Buenos Aires, Argentina; 2 Departamento de Bioquímica Médica y Biología Molecular. Hospital Universitario Virgen Macarena, Facultad de Medicina, Universidad de Sevilla, Sevilla, España; 3 Hospital Nacional Profesor Alejandro Posadas, Buenos Aires, Argentina; 4 Servicio de Ginecología y Obstetricia, Hospital Universitario Virgen Macarena, Sevilla, España; 5 Instituto de Biología y Medicina Experimental (IBYME), Buenos Aires, Argentina; John Hunter Hospital, Australia

## Abstract

Pleiotropic effects of leptin have been identified in reproduction and pregnancy, particularly in the placenta, where it works as an autocrine hormone. In this work, we demonstrated that human chorionic gonadotropin (hCG) added to JEG-3 cell line or to placental explants induces endogenous leptin expression. We also found that hCG increased cAMP intracellular levels in BeWo cells in a dose-dependent manner, stimulated cAMP response element (CRE) activity and the cotransfection with an expression plasmid of a dominant negative mutant of CREB caused a significant inhibition of hCG stimulation of leptin promoter activity. These results demonstrate that hCG indeed activates cAMP/PKA pathway, and that this pathway is involved in leptin expression. Nevertheless, we found leptin induction by hCG is dependent on cAMP levels. Treatment with (Bu)_2_cAMP in combination with low and non stimulatory hCG concentrations led to an increase in leptin expression, whereas stimulatory concentrations showed the opposite effect. We found that specific PKA inhibition by H89 caused a significant increase of hCG leptin induction, suggesting that probably high cAMP levels might inhibit hCG effect. It was found that hCG enhancement of leptin mRNA expression involved the MAPK pathway. In this work, we demonstrated that hCG leptin induction through the MAPK signaling pathway is inhibited by PKA. We observed that ERK1/2 phosphorylation increased when hCG treatment was combined with H89. In view of these results, the involvement of the alternative cAMP/Epac signaling pathway was studied. We observed that a cAMP analogue that specifically activates Epac (CPT-OMe) stimulated leptin expression by hCG. In addition, the overexpression of Epac and Rap1 proteins increased leptin promoter activity and enhanced hCG. In conclusion, we provide evidence suggesting that hCG induction of leptin gene expression in placenta is mediated not only by activation of the MAPK signaling pathway but also by the alternative cAMP/Epac signaling pathway.

## Introduction

The major role of the placenta is to establish a crosstalk between maternal and fetal circulations. In addition, the placenta works as an endocrine tissue that produces steroids, peptide hormones, growth factors and cytokines that are crucial for the establishment and maintenance of pregnancy. Several cytokines and growth factors, such as leptin, are known to influence trophoblast migration, proliferation and invasion [1].

Leptin, the product of the LEP gene, is a small non-glycosylated pleiotropic peptide of 146 aminoacid residues (16 kDa), firstly found to be secreted by adipose tissue [2], with the function of modulation of satiety and energy homeostasis [3]. Compelling evidence also implicated leptin in reproductive functions such as the regulation of fertility, ovarian function, oocyte maturation, embryo development and implantation [4], [5], [6]. The synthesis and secretion of leptin as well as its functional receptors by trophoblast cells have been widely demonstrated [7], [8], suggesting that leptin may act through a paracrine or autocrine mechanism. In this way, previous studies have demonstrated the interactions between leptin and some placental hormones, implicating leptin as a modulator of placental endocrine function [9]. Moreover, leptin stimulates the process of proliferation and protein synthesis, and inhibits apoptosis [10], [11], [12], [13] in human trophoblastic cells.

Deregulation of leptin metabolism and/or leptin function in the placenta may be implicated in the pathogenesis of various disorders during pregnancy, such as recurrent miscarriage, gestational diabetes, intrauterine growth restriction, and preeclampsia [14], [15].

Placental leptin production is strictly regulated and there are differences between the regulation of transcription of human placental and adipose leptin [16]. In fact, the human leptin gene has an enhancer located at −1.9 kb that is activated by a placental-specific transcription factor [17]. In this context, we have previously reported that human chorionic gonadotropin (hCG), a key hormone in pregnancy, upregulates placental leptin [18].

HCG mediates its action through the LH/hCG receptor, and its major function is to maintain the progesterone production of corpus luteum during early pregnancy. Binding of hCG to its receptor generates, in the classical response, an increase in cyclic adenosine monophosphate (cAMP) concentration and a consequent activation of protein kinase A (PKA) (29). We previously observed that (Bu)_2_cAMP not only did not enhance hCG effect but also inhibited hCG dependent leptin expression in placental cells [18]. The LH/hCG receptor has also been shown to mediate activation of the mitogen-activated protein kinase (MAPK) (56, 57), Janus kinase and PI3K signaling pathways (58). Certainly, we demonstrated that hCG treatment specifically activates MEK and extracellular signal-regulated kinase 1/2 (ERK1/2) phosphorylation in placental cells and that this signal transduction pathway is involved in hCG leptin up-regulation [18].

On the other hand, in a previous work we reported that the expression of placental leptin is also regulated by cAMP [19]. Increases in intracellular cAMP classically lead to the activation of PKA, which phosphorylates intracellular substrates, including the cAMP response element binding protein (CREB) [20], [21]. However, several reports have provided evidence that cAMP affects some cellular processes independently of PKA [22], [23], [24]. Indeed, in that previous work, we demonstrated that leptin up-regulation by cAMP involves a crosstalk between PKA and MAPK signaling pathways [19]. It was also shown that hCG enhanced leptin expression in placental cells involving a crosstalk between cAMP and p38 pathways [25]. In many cellular activities cAMP signaling has others mediators besides PKA, including cyclic nucleotide-gated ion channels, and exchange protein directly activated by cAMP (Epac) [26]. Interestingly, the two Epac1 and Epac2 isoforms were identified as cAMP-binding proteins with guanine nucleotide exchange factor (GEF) activities for the small GTPases, Rap1 and Rap2 [27]. Upon cAMP binding, Epac proteins undergo a conformational change, which relieves the autoinhibition and then activates Rap proteins to regulate numerous cellular actions [28].

It was demonstrated that the cAMP pathway can regulate ERK signaling through several distinct mechanisms providing important crosstalk between hormones and growth factor signaling. A model that explains the activation of ERK by cAMP includes the involvement of either Rap1 or Ras, and might include PKA independent actions of cAMP [29].

The role of Epacs in Rap1 activation has been supported by studies using 8-CPT-2Me-cAMP, a cAMP analogue that retains a high affinity for Epac but does not activate PKA [30]. Epac-mediated cAMP signaling is involved in cellular functions such as cell differentiation, secretion/exocytosis, cell adhesion and cell–cell junctions [31].

It was seen that in first and second trimester placenta, both Epac1 and Epac2 were expressed in villous syncytiotrophoblast (STB), cytotrophoblast (CTB), stroma, blood vessels and extravillous trophoblast (EVT). In term placenta, Epac1 and Epac2 were mainly distributed in the STB layer, EVT and blood vessels. Interestingly, Epac1 and Epac2 were localized at the plasma membrane and in the cytoplasm of BeWo cells, respectively [32]. The physiological significance of Epac expression on trophoblast function is not completely known. In the last few years, the Epac signaling pathway was associated with the cAMP-mediated functional differentiation and syncytialization of human trophoblasts [32].

Since hCG and cAMP play a critical role in the control of numerous placental hormones and seem to mediate leptin gene expression in placenta, and given that multiple effectors can be involved in the transduction of cAMP signaling, we reasoned that leptin expression may be regulated through different effectors of the cAMP signaling cascade. In the present work, we hypothesized that placental leptin, as a key molecule of the implantation and pregnancy, is finely regulated by hCG, a central hormone in pregnancy, and that this regulation depends on a delicate balance between MAPK and Epac/cAMP signaling pathways. Thus, we aimed to investigate the mechanisms governing the regulation of leptin expression by hCG and cAMP in human placenta and to unravel the signaling pathways involved.

Our study demonstrates that hCG induces leptin expression in placenta through the MAPK pathway and cAMP stimulation. At the same time, the role of cAMP as a second messenger of hCG would be PKA independent and involves the activation of the alternative cAMP/Epac pathway.

## Materials and Methods

### Ethics Statement

Written informed consent was obtained from all subjects and all study procedures were approved by ethical review committees at the Virgen Macarena University Hospital and the Alejandro Posadas National Hospital (Bioethics Comitte “Dr. Vicente Federico del Giudice”).

### Cell Culture and Treatments

The human choriocarcinoma cell lines BeWo and JEG-3 were purchased from the American Type Culture Collection (ATCC, Rockville, MD). Cells were grown in DMEM-F12 (Invitrogen, Carlsbad, CA) supplemented with 10% fetal bovine serum (FBS), 100 U/ml penicillin, 100µg/ml, streptomycin, 2 mM glutamine (Invitrogen), and 1 mM sodium pyruvate (Sigma Chemical Co., St. Louis, MO) at 37 C in 5% CO_2_. To test the effect of cAMP, the cAMP analogue dibutyryl cAMP [(Bu)_2_cAMP] (1 µM to 1 mM) (Sigma Chemical Co.) was used to facilitate cell entrance. The effect of recombinant hCG (Sigma Chemical Company, St. Louis, MO) was tested at different doses (5 IU hCG/ml to 100 IU hCG/ml). In experiments designed to analyze the signal transduction pathways involved in hCG stimulation of leptin, the cell-permeable adenylyl cyclase inhibitor SQ22,536 (100 µM), the selective inhibitor of cAMP-dependent protein kinase (PKA) H89 (10 µM), the MAPK kinase (MEK) inhibitor PD98059 (50 µM) and the selective cAMP analogue that stimulates Epac, 8CPT-2me-cAMP (10 µM) (Sigma Chemical Co.) were used. Inhibitors were added 30 min before hCG or cAMP analogues treatment, except in experiments performed to determine protein phosphorylation, in which the inhibitors were added 10 min before treatments. All treatments were performed in DMEM-F12 media supplemented with 1% FBS unless indicated. Serum present in the media of incubation was reduced from 10 to 1% to lower nonspecific effects.

### Placental Explants Collection and Processing

Human placentas (n = 9) were obtained after cesarean section or vaginal delivery after normal term pregnancies and immediately suspended in ice-cold PBS and transported to the laboratory, where they were washed two to three times in sterile PBS to remove excess blood. Villous tissue free of visible infarct, calcification or hematoma was sampled from at least five cotyledons at a distance midway between the chorionic and basal plates. These core parts of cotyledons were cut into multiple cubic segments (10 to 15 mg wet weight) and thoroughly rinsed with cold Hanks’ medium pH 7.4 (137 mM NaCl, 5 mM KCl, 1 mM CaCl_2_, 1 mM MgSO_4_, 0.3 mM Na_2_HPO_4_, 0.4 mM KH_2_PO_4_, and 4 mM NaHCO_3_). None of the donor patients suffered from anomalous pregnancy. This study was approved by the patient’s written consent and by the local ethical committee.

### Treatments of Placental Explants

Placental explants were randomly distributed in tubes containing 1 ml of DMEM-F12 medium (n = 1 explant/tube, four replicates per treatment). Placental explants were maintained in a shaking water bath at 37°C during 5 min to equilibrate temperature, pre-incubated during 30 min when indicated with 50 µM PD98059, 10 µM H89 and incubated in the same medium supplemented or not with hCG (100 IU/ml) and/or 10 µM Cpt-O-Me or 10 µM (Bu)_2_cAMP during 4 h for leptin expression analysis and 15 min for phosphorylation experiments. Explants were removed from the bath, centrifuged for 2 min at 2000 g at 4°C and resuspended in 500 µl of lysis buffer (1×PBS, 1% Nonidet P-40, 0.5% sodium deoxycholate, 0.1% sodium dodecyl sulfate (SDS), and 10 mg/ml phenylmethanesulfonyl fluoride (PMSF)) during 30 min at 4°C on an orbital shaker and later centrifuged at 10000 g for 20 min. Supernatants were analyzed by Western blot.

For real-time PCR, after thoroughly washing with phosphate buffer saline, the tissues were immediately frozen at 80°C and stored until extraction of total RNA.

### Western Blot Analysis

Cells were seeded at 50–60% confluence in DMEM-F12 medium supplemented with 10% FBS. Each treatment was performed in the same media supplemented with 1% FBS during 3 days for leptin immunoblot or during 10 min for protein phosphorylation determinations. Total cell lysates were prepared in lysis buffer. The lysates were centrifuged at 10,000 g for 10 min to remove cellular debris. The protein concentration of the supernatant was determined by the Bradford staining method [33], with BSA as standard. Lysates were mixed with Laemmli’s sample buffer containing 2% sodium dodecyl sulfate and 30 mM β-mercaptoethanol, boiled for 5 min, resolved by SDS-PAGE on a 12% gel, and electrophoretically transferred to a nitrocellulose membrane (Hybond; Amersham Pharmacia Biotech, Piscataway, NJ) thereafter. Membranes were equilibrated in 1X PBS, and nonspecific binding sites were blocked by 5% nonfat milk in PBS at room temperature for 1 h. The membranes were then immunoblotted with polyclonal rabbit antihuman leptin Y20 (1∶1000) (Santa Cruz Biotechnology, Inc., Santa Cruz, CA) or with polyclonal rabbit antiphospho-ERK 1/2 (Thr202/Tyr204) (1∶3000) (New England Biolabs). Loading controls were performed by immunoblotting the same membranes with polyclonal rabbit anti-β-actin (1∶5000) (Sigma Chemical Co.), or with polyclonal rabbit anti total-ERK 1/2 (1∶3000). The antibodies were detected using horseradish peroxidase-linked goat anti-rabbit IgG (1∶10000) (Santa Cruz Biotechnology, Inc.) and visualized by the Amersham Pharmacia enhanced chemiluminescence signaling system and a Bio-Imaging Analyzer Fujifilm LAS-1000 (Fuji Photo Film Co., Ltd., Tokyo, Japan). Quantification of protein bands was determined by densitometry using Image J ink 1.45 program (National Institute of Health, Bethesda, MD, USA).

### Plasmids

The luciferase (*Luc*) reporter constructs are based on pGL-3 basic vector. They were all kindly provided by Oksana Gavrilova [17]. pRSV-β-gal contains the β-galactosidase gene under the control of the Rous sarcoma virus (RSV) promoter and was used to normalize the efficiency of individual transfections. pMtC-α is a 5.4-kb expression vector plasmid containing the cDNA for the α isoform of the mouse cAMP-dependent protein kinase (PKA) catalytic subunit [34]. pMt-REV is a 7.6-kb expression vector that contains a dominant negative mutant cDNA of the mouse PKA regulatory I-subunit (PKI) inserted between the mouse metallothionein-promoter and the polyadenylation signal sequence of the human GH gene [35].

Plasmids pMT2-HA-Rap1A contained the coding sequence for murine Rap1A [36]; pMT2-HA-Epac, pMT2-HA-Rap1GAP and expression vector for negative mutant of CREB, pCREBM1, were a generous gift of Dr O. Coso (Department of Physiology and Molecular Biology, FCEN, UBA, Buenos Aires, Argentina). Plasmid CREBM1 contains a conservative serine-to-alanine substitution at position 133 that destroys the PKA phosphorylation site [37]. Plasmid pGL3-CRE-Luc containing the CRE element cloned next to the luciferase gene was kindly provided by Adalí Pecci (Department of Biological Chemistry, FCEN, UBA, Buenos Aires, Argentina).

In experiments using expression plasmids, the empty vectors were used as controls. To perform transient transfection assays, plasmids were purified using the Maxipreps Wizard kit (Promega Corp., Madison, WI), and the concentration of DNA was estimated spectrophotometrically.

### Transient Transfection Experiments

For transient transfection experiments, BeWo cells were plated at a density of 2.5×10^5^ cells/ml onto six-well dishes containing 2 ml of DMEM-F12 plus 10% FBS. Cells were incubated for 24 h. Medium was replaced, and transfection of cells was performed according to the standard liposome-mediated method. To determine the sensitivity of the method in this cell type, a standard dose of reporter plasmid vs. light emission was performed (data not shown). Typically, 5 µg of the Luc reporter and 5 µg of pRSVβ-gal internal control construct were transfected using 5 µl of LipofectAMINE (Life Technologies, Inc., Gaithersburg, MD). The medium was replaced after 5 h with DMEM-F12 1% FBS with the addition of the different effectors. Transfection analysis was performed by duplicate in each of at least three independent experiments.

### Assays for Luc and β-galactosidase Activities

Luc activity in cell lysates was measured using the Luc Assay System (Promega Corp.). Cells were washed with PBS and harvested 72 h after transfection using 50 µl of lysis buffer. Cell extracts were centrifuged and 30 µl of the supernatant was mixed with 50 µl of Luc assay buffer. Luc activity was measured with the GloMax-Multi+ Microplate Multimode Reader luminometer (Promega Corp). β-Galactosidase activity was assayed using 1 mg of onitrophenyl-D-galactopyranoside (AmResco, Solon, OH) as the substrate in buffer Z (60 mM Na_2_HPO_4_, 40 mM NaH_2_PO_4_,10 mM KCl, 1 mM MgSO_4_, and 0.07% β-mercaptoethanol) and incubated at 37°C until yellow staining. The product was determined by absorption at 420 nm. This value was used to correct variations in transfection efficiency. Luc results were calculated as the ratio of Luc activity per unit of β-galactosidase activity. Duplicate samples were analyzed for each data point.

### Quantitative Real-Time RT-PCR (qRT-PCR) Assay

Abundance of leptin mRNA was determined by qRT-PCR. Total RNA was extracted from JEG-3 or placental explants using TRISURE reagent, according to the manufacturés instructions (Bioline Co., Essex, UK). Concentration and purity of the isolated RNA were estimated spectrophotometrically at 260 and 280 nm. For cDNA synthesis, 5 µg of total RNA was reverse transcribed at 50°C during 1 h using the Transcriptor first Strand cDNA synthesis Kit (Roche, Indianapolis, IN). Quantitative real- time PCR was performed using the following primers based on the sequences of the National Center for Biotechnology Information GenBank database: leptin: forward, 5′GAACCCTGTGATTCTT3′; reverse, 5′CCAGGTCGTTATTTGG3′; and cyclophilin: forward, 5′CTTCCCCGATACTTCA 3′; reverse,5′TCTTGGTGCTACCTC3′. Quantitative RT-PCR Master Mix Reagent kit was obtained from Roche (Fast Start universal SYBR Green), and PCRs were performed on a Chromo 4 DNA Engine (Bio-Rad, Hercules, CA). A typical reaction contained 10 µM of forward and reverse primer, 3 µl of cDNA, and the final reaction volume was 25 µl. The reaction was initiated by preheating at 50°C for 2 min, followed by heating at 95°C for 10 min. Subsequently, 41 amplification cycles were carried out as follows: denaturation 15 sec at 95°C and 1 min annealing and extension at 58°C. The threshold cycle (CT) from each well was determined by the Opticon Monitor 3.1.32 Program (BioRad Laboratories Inc). Relative quantification was calculated using the 2^−ΔΔCT^ method [38]. For the treated samples, evaluation of 2^−ΔΔCT^ indicates the fold change in gene expression, normalized to a housekeeping gene (cyclophilin), and relative to the untreated control. Melting curve analysis was performed to confirm specificity of amplification. Reaction mixtures without reverse transcriptase or RNA were run in parallel to ensure the absence of sample contamination.

### Intracellular cAMP Determination

BeWo cells were incubated in sterile 96-well plate with a seeding density of 10000 cells per well. Cells were cultured in DMEM-F12 10% FBS during 24 h. Medium was replaced by DMEM-F12 1% SFB and cells were treated with hCG (0–500 IU/ml) for 72 h. Then, cAMP-Glo assay kit was used according to manufacturer’s instruction (Promega Corp.). Luminescence was measured using the GloMax-Multi+ Microplate Multimode Reader luminometer (Promega Corp).

### Data Analysis

For Western blots analysis, representative images of at least three independent experiments are shown along with quantification of immunoreactive bands. Quantitative RT-PCR experiments were repeated separately at least three times to ensure reproducible results. Transient transfection experiments were repeated at least three times and each treatment performed by duplicates. Results are expressed as the meanSD. The statistical significance was assessed by ANOVA followed by Bonferroni’s multiple comparison post hoc test and was calculated using the GraphPad Instat computer program (GraphPad, San Diego, CA). A P value less than 0.05 was considered statistically significant.

## Results

### HCG Stimulates Leptin Expression and Increases cAMP Levels in placenta

**Figure 1:**
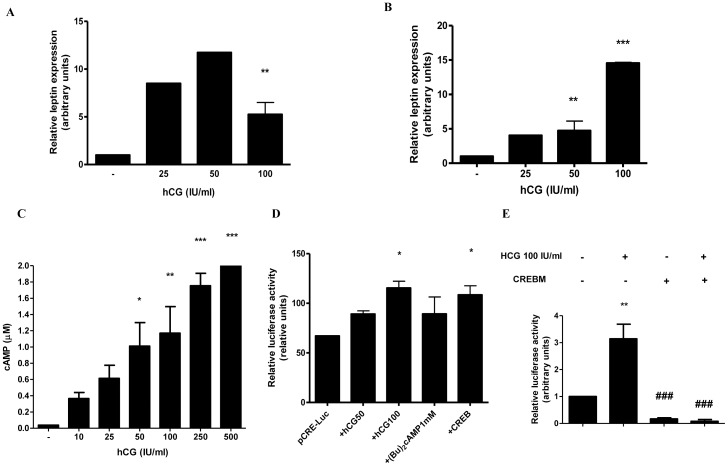
hCG stimulates leptin mRNA expression and enhances cAMP levels in placenta. (A) JEG-3 cells (1×10^6^ cells) were plated in complete DMEM-F12 media supplemented with 1% FBS and incubated during 3 days with different doses of hCG (IU/ml). (B) Placental explants were obtained as indicated in Materials and Methods and treated with increasing hCG concentrations. In (A) and (B), total RNA was extracted as described in Material and Methods. Leptin mRNA was quantified by real time RT-PCR. Cyclophilin was used as internal standard. (C) BeWo cells (1×10^5^) were seeded in 96-well plate and treated during 24 h with increasing concentrations of hCG, as indicated. cAMP-Glo assay kit was used to measure intracellular cAMP concentration. (D) Cells were transiently transfected with pCre-Luc plasmid construction and treated with hCG, (Bu)_2_cAMP or cotransfected with CREB, as indicated, during 72 h in DMEM-F12 media supplemented with 1% FBS. Luciferase activity was measured in cellular extracts and normalized to β-galactosidase activity. Activity obtained with empty vector (PGL-3 basic vector) was set as a control. (E) BeWo cells were transiently transfected with pL1951 and treated with 100 IU/ml hCG and/or cotransfected with CREBM plasmid. Cells were incubated during 72 h in DMEM-F12 1% FBS media. Luciferase activity was measured in cellular extracts and normalized to β-galactosidase activity. Activity obtained with empty vector (PGL-3 basic vector) was set as a control. Results shown are from a representative experiment and are expressed as means ± S.E.M. for three independent experiments performed in duplicates. *p<0.05, **p<0.01, ***p<0.001 vs control; ###p<0.001 vs hCG treatment.

The choriocarcinoma cell lines BeWo and JEG-3 were used as models for trophoblastic cells as previously reported [7], [39]. Previous results have demonstrated that leptin and leptin receptor are expressed in these cell lines, suggesting that leptin is probably exerting both paracrine and autocrine effects [11]. We have previously shown that hCG and cAMP stimulate leptin expression in BeWo and JEG-3 cells, as well as in placental explants [18], [19]. In this regard, we aimed to demonstrate that hCG stimulates leptin expression not only at the protein but also at the mRNA level. Figure 1A shows that hCG (25–100 IU/ml) enhanced leptin mRNA expression in JEG-3 cells, measured by *q*RT-PCR. Maximal effect was achieved at 50 IU/ml, reaching a 11,7-fold increase. The same effect was observed in placental explants (Fig. 1B), where hCG significantly stimulated leptin mRNA expression, reaching a 14,5-fold maximal increase at 100 IU/ml. These results reinforce the notion that hCG has a role in regulating leptin expression. On the other hand, we have previously observed that treatment of trophoblastic cells with (Bu)_2_cAMP causes a complete loss of hCG leptin induction [18]. For this reason, we aimed to determine if hCG could enhance cAMP levels in the placenta. As seen in Fig. 1C, treatment with hCG caused an increase in cAMP levels in BeWo cells, in a dose dependent manner. Maximal effect was achieved at 500 IU/ml hCG, a dose that elevated intracellular cAMP concentration to 3,8 µM. To further confirm activation of the cAMP pathway by hCG, we performed a transient transfection assay with a vector containing tandem CRE elements fused to the Luc reporter gene (pCRE-Luc). BeWo cells were treated with hCG (50 or 100 IU/ml), 1 mM (Bu)_2_cAMP or cotransfected with the expression plasmid for the transcription factor CREB. Results are shown in Fig. 1D. HCG stimulated CRE elements activity, reaching a 1,72-fold induction with 100 IU/ml of hCG. This stimulation was even higher than the stimulation caused by cAMP or by cotransfection with CREB. To establish a link between hCG and CREB transactivation on the leptin promoter, we performed a cotransfection with pL1951 and an expression plasmid for a mutant CREB (CREBM1), containing a conservative serine-to-alanine substitution at position 133 that destroys the PKA phosphorylation site. Results in Figure 1E show that CREBM1 was completely unable to activate transcription of leptin promoter both in the presence or absence of hCG. Cotransfection with CREBM1 caused a significant inhibition of hCG stimulation of leptin promoter activity, with a 112-fold reduction compared with hCG treatment. Taken together, these results demonstrate that hCG is able to increase cAMP levels in trophoblastic cells and this effect probably leads not only to activation of CREB protein but also to the stimulation of the transcription of different genes trough CRE elements.

### High cAMP Levels Interfere with hCG Stimulation of Leptin Expression

**Figure 2:**
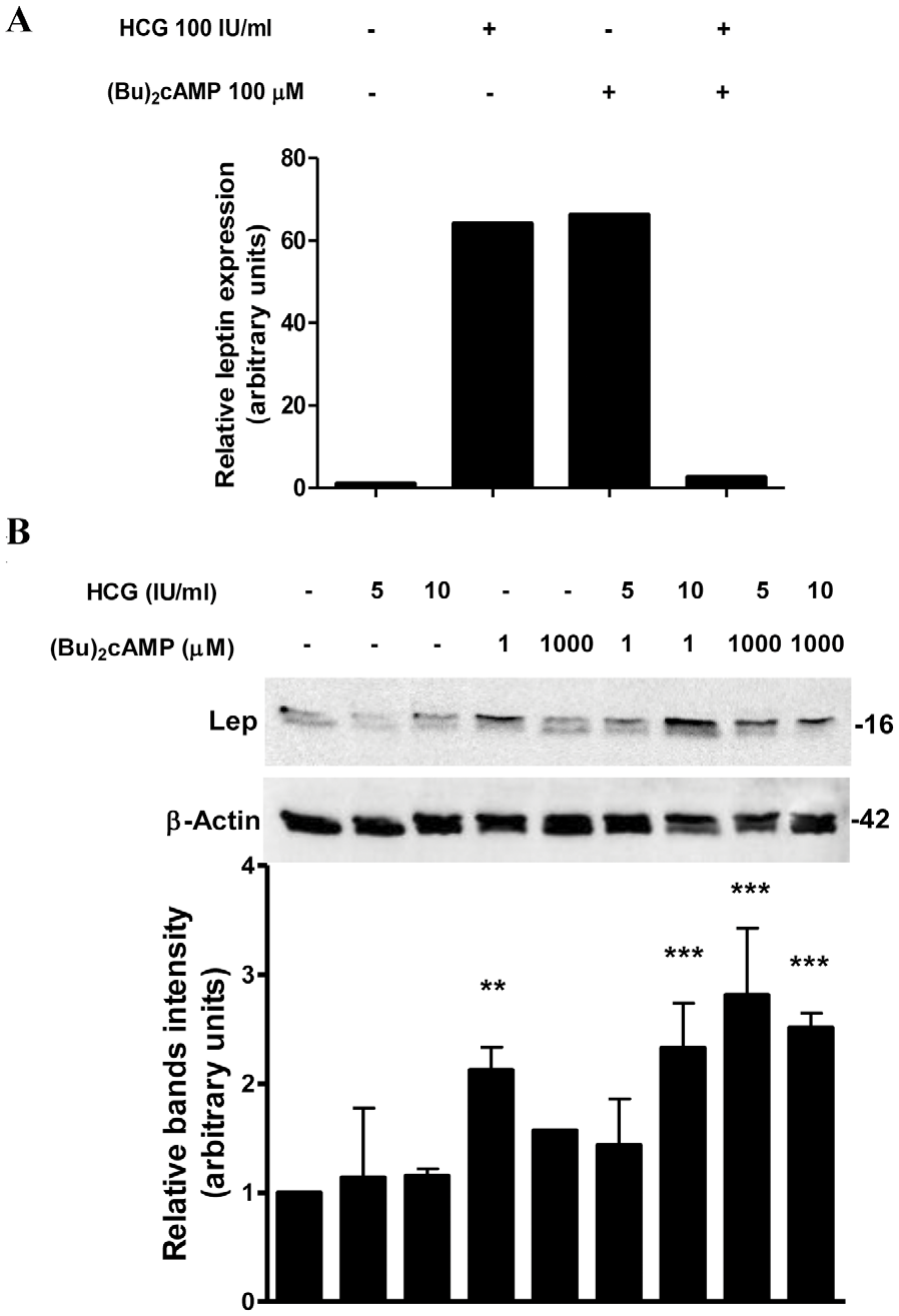
cAMP induces leptin stimulation by hCG at low hormone concentrations. (A) Placental explants were processed as previously described and treated with increasing hCG and/or (Bu)_2_cAMP concentrations during 4 h. Total RNA was extracted as described in Material and Methods. Leptin mRNA was quantified with real time RT-PCR. Cyclophilin was used as internal standard. (B) BeWo cells (1×10^6^ cells) were plated in complete DMEM-F12 media supplemented with 1% FBS and incubated during 3 days with different doses of hCG (IU/ml) and/or (Bu)_2_cAMP (µM), as indicated. Cell extracts were prepared as indicated in Materials and Methods. Proteins were separated on SDS-PAGE gels and leptin expression was determined by Western-blot. Molecular weights were estimated using standard protein markers. Loading controls were performed by immunoblotting the same membranes with anti-β-actin. Bands densitometry is shown in lower panels. Molecular weight (kDa) is indicated at the right of the blot. Representative results from three replicates are shown. **p<0.01, ***p<0.001.

Previous data have shown that treatment with hCG (50 or 100 IU/ml) in combination with (Bu)_2_cAMP causes a complete loss of hCG induction of leptin expression in placental cells [18]. These results were observed both at the transcriptional and protein levels. In order to measure cAMP effect on hCG enhanced leptin mRNA expression, we performed quantitative RT-PCR in placental explants. As expected, hCG and cAMP increased leptin mRNA levels 64 and 66-fold respectively (Fig. 2A). When hCG treatment was combined with cAMP, leptin mRNA expression was inhibited 62-fold relative to hCG treatment.

Some authors have shown that hCG receptor can be positively or negatively regulated depending on cAMP concentration and time of exposure [40]. This evidence raises the possibility that the observed inhibition of hCG leptin induction, may be due to high intracellular cAMP levels generated by hCG treatment (Fig0 1C) combined with exogenously added (Bu)_2_cAMP. In this context, we determined leptin expression by Western blot in BeWo cells treated with low and non stimulatory hCG concentrations in combination or not with (Bu)_2_cAMP. Under these experimental conditions, cAMP treatment led to a significant increase in hCG leptin induction (Fig. 2B). Maximal effect was achieved at 1 mM (Bu)_2_cAMP with a 2,81-fold increase. As expected, treatment with 5 or 10 IU/ml hCG alone, had no effect on leptin expression. Taken together, these results demonstrate that high cAMP concentrations could lead to inhibition of the hCG stimulatory effect.

### The hCG Stimulatory Effect on Leptin Expression is Blocked by PKA Activation

**Figure 3:**
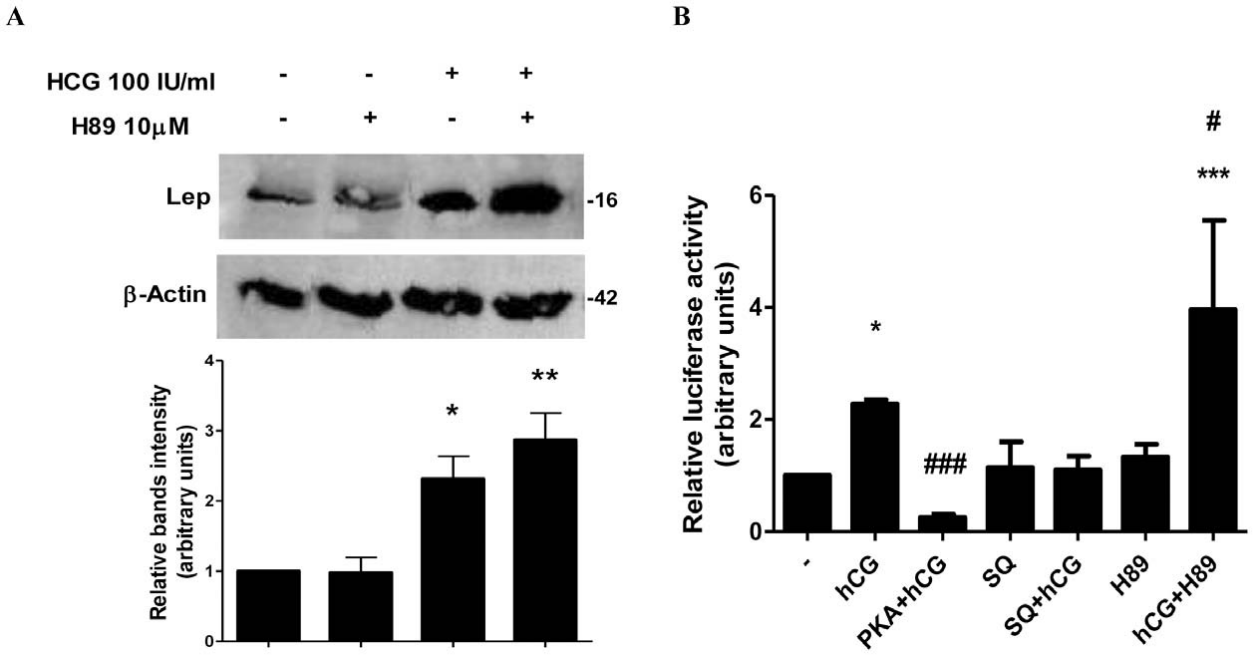
PKA blocks hCG stimulation of leptin. (A) BeWo cells were incubated during 3 days with hCG and/or H89, as indicated. Extracts from cells were prepared as previously described and loaded in a 12% SDS-PAGE. Leptin expression was determined by Western-blot. Loading controls were performed by immunoblotting the same membranes with anti-β-actin. Bands densitometry is shown in lower panels. Molecular weight (kDa) is indicated at the right of the blot. Representative results from three replicates are shown. (B) BeWo cells were transiently transfected with pL1951 and treated with 100 IU/ml hCG, 10 µM H89 and/or 100 µM SQ, or cotransfected with a plasmid expressing the catalytic subunit of PKA (PKA) (1 µg/ml) (C), or with a dominant negative mutant of the regulatory subunit of PKA (PKI) (1 µg/ml). Cells were incubated during 72 h in DMEM-F12 1% FBS media. Luciferase activity was measured in cellular extracts and normalized to β-galactosidase activity. Activity obtained with empty vector (PGL-3 basic vector) was set as a control. Results are expressed as mean ± S.E.M. for three independent experiments. *p<0.05, **p<0.01, ***p<0.001 vs. control; #p<0.05, ###p<0.001 vs. hCG treatment.

PKA activation is the classical pathway activated by cAMP when the levels of this nucleotide rise [41]. Therefore, we next investigated the involvement of PKA on leptin induction by hCG. BeWo cells were treated with 100 IU/ml hCG in the presence or absence of 10 µM H89, a specific PKA inhibitor. Leptin expression was measured by Western blot analysis (Fig. 3A). As previously seen, 100 IU/ml hCG produced a 2,32-fold induction of leptin expression. This effect was even higher when cells were pretreated with 10 µM H89, reaching a 2,87-fold induction over control. We next decided to examine the effect of PKA activation on hCG leptin induction at the transcriptional level. BeWo cells were transiently transfected with pL1951 Luc reporter construct and treated with hCG, H89 or SQ (a specific adenilyl cyclase inhibitor), and/or cotransfected with the expression plasmid for the catalytic subunit of PKA, as indicated. As displayed in Fig. 3B, treatment with hCG significantly stimulated leptin promoter activity and this effect was enhanced 2-fold upon combination with H89. Moreover, hCG effect on leptin expression was inhibited 8,7-fold when cells overexpressed the catalytic PKA subunit. Treatment with SQ alone or in the presence of hCG showed no effect on leptin expression. All together, these results suggest that the PKA signaling pathway is not involved in the mechanisms regulating hCG leptin induction. Furthermore, the observed cAMP inhibition of hCG action on placental leptin could be mediated by PKA activation.

### PKA Inhibits the hCG Activation of MAPK Signaling Pathway

**Figure 4:**
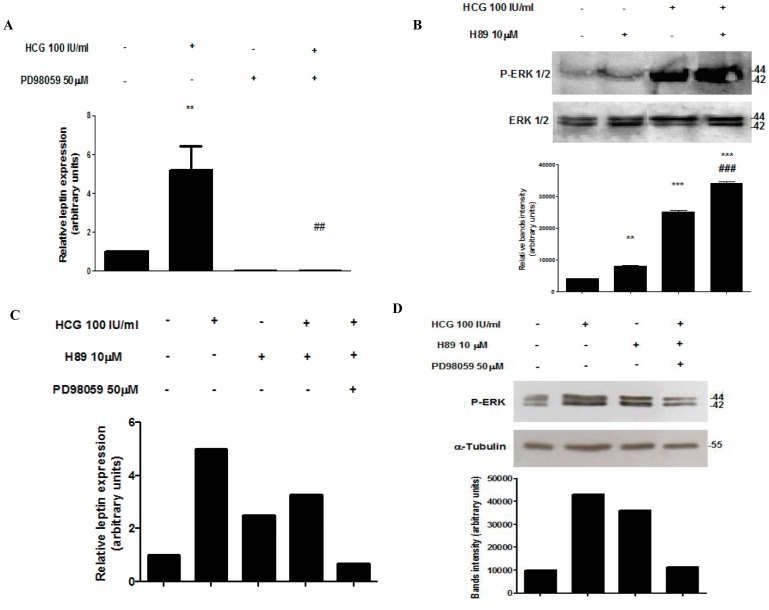
PKA inhibits ERK activation by hCG. (A) JEG-3 cells (1×10^6^ cells) were plated in complete DMEM-F12 media supplemented with 1% FBS and incubated during 3 days with hCG (IU/ml) and/or PD98059. Total RNA was extracted as described in Material and Methods. Leptin mRNA was quantified by real time RT-PCR. Cyclophilin was used as internal standard. Results are expressed as mean ± S.E.M. for three independent experiments performed in triplicates. (B) BeWo cells were incubated for 15 min with hCG and/or H89 as indicated. Extracts from cells were prepared as previously described and loaded in a 12% SDS-PAGE. ERK 1/2 phosphorylation was determined by Western blot. Total ERK 1/2 protein level in extracts was determined as loading control. Molecular weights were estimated using standard protein markers. Bands densitometry is shown in lower panel. Results shown are from a representative experiment and are expressed as means ± S.E.M. for three independent experiments **p<0.01 ***p<0.001 vs control; ###p<0.001 vs hCG treatment. (C) Placental explants were processed as previously described pre-incubated during 30 min with 50 µM PD98059 and/or 10 µM H89 and treated with 100 IU/ml hCG during 4 h (C) or 15 min (D). (C) Total RNA was extracted as described in Material and Methods. Leptin mRNA was quantified by real time RT-PCR. Cyclophilin was used as internal standard. (D) Extracts were prepared as previously described and loaded in a 12% SDS-PAGE. ERK 1/2 phosphorylation was determined by Western blot. Total ERK 1/2 protein level in extracts was determined as loading control. Molecular weights were estimated using standard protein markers. Bands densitometry is shown in lower panel.

We have previously demonstrated that hCG stimulates the MAPK/ERK signaling transduction pathway in placental cells [18]. Other authors have also reported such ability of LH/hCG receptor in different tissues [42], [43], [44]. In this way, we aimed to investigate the effect of MAPK inhibition on leptin mRNA expression in trophoblastic cells. JEG-3 cells were treated with hCG or/and PD98059, a pharmacologic inhibitor that blocks MEK’s ability to activate ERKs. Leptin mRNA was measured by *q*RT-PCR. Results shown in Fig.4A confirmed that hCG significantly stimulated leptin mRNA and that cotreatment with MEK inhibitor completely blocked this induction. Based on these results, we hypothesized that cAMP inhibition of hCG effect on leptin expression could be partially due to the impairment of MAPK signaling pathway by PKA.

To this end, we evaluated ERK activation in BeWo cells treated with hCG in the presence or not of H89. ERK 1/2 phosphorylation was assessed by Western blot. As seen in Fig. 4B, hCG induced a 6-fold increase in ERK phosphorylation. When hCG was combined with H89, the activation of ERK reached an 8-fold increase compared to the control and a 4-fold induction above hCG treatment. Moreover, when we treated placental explants with hCG in combination with H89 and PD98059, we observed an inhibition in hCG stimulation of leptin expression, measured by real time PCR (Fig. 4C). Results shown in Fig. 4D demonstrates that phosphorylation of ERK 1/2 is inhibited when placental explants are treated with hCG plus H89 and PD98059. The effect of MAPK inhibition prevails over the inhibition of PKA, suggesting that the MAPK signaling pathway would be the main pathway involved on the stimulatory effect of hCG on leptin expression.

Taking together, these results demonstrate that hCG stimulates the MAPK pathway and that cAMP/PKA pathway activation is probably blocking this effect.

### The Alternative cAMP/Epac Signaling Pathway is Involved in Placental Leptin Induction by hCG

**Figure 5:**
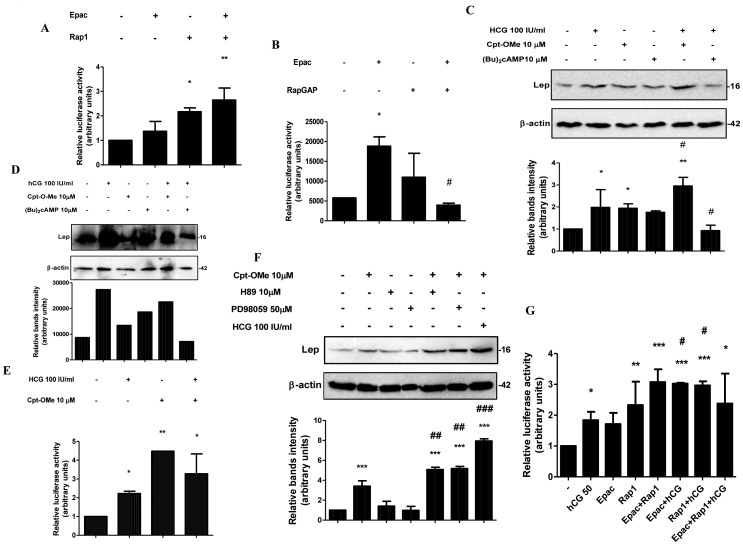
hCG induces leptin expression through the cAMP/Epac alternative signaling pathway. (A) BeWo cells were transiently cotransfected with pL1951 and Epac (1 µg/ml) and/or Rap1b (1 µg/ml) proteins expression plasmids. (B) BeWo cells were transiently cotransfected with pL1951 Epac (1 µg/ml) and/or RapGAP (1 µg/ml) proteins expression plasmids. (C) and (E) BeWo cells (1×10^6^ cells) were plated in complete DMEM-F12 media supplemented with 1% FBS and incubated during 3 days with different doses of Cpt-OMe, hCG, (Bu)_2_cAMP, and H89, as indicated. Cell extracts were prepared as indicated in Materials and Methods. Proteins were separated on SDS-PAGE gels and leptin expression was determined by Western-blot. Molecular weights were estimated using standard protein markers. Loading controls were performed by immunoblotting the same membranes with anti-β-actin. Bands densitometry is shown in lower panels. Molecular weight (kDa) is indicated at the right of the blot. Representative results from three replicates are shown. (D) Cells were transfected with pL1951 plasmid construction and treated with hCG and/or Cpt-OMe, as indicated. (F) BeWo cells were cotransfected with pL1951 and/or Epac and Rap1b and treated with hCG (IU/ml). In (A), (B), (D) and (F) cells were incubated during 72 h in DMEM-F12 1% FBS media. Luciferase activity was measured in cellular extracts and normalized to β-galactosidase activity. Activity obtained with empty vector (PGL-3 basic vector) was set as a control. Results are expressed as mean ± S.E.M. for three independent experiments. *p<0.05, **p<0.01, ***p<0.001 vs. control; #p<0.05, ##p<0.01, ###p<0.001 vs. hCG treatment. (G) Placental explants were processed as previously described and treated with 100 IU/ml hCG and/or 10 µM Cpt-O-Me or 10 µM (Bu)_2_cAMP as indicated during 4 h. Proteins were separated on SDS-PAGE gels and leptin expression was determined by Western-blot. Molecular weights were estimated using standard protein markers. Loading controls were performed by immunoblotting the same membranes with anti-β-actin. Bands densitometry is shown in lower panels. Molecular weight (kDa) is indicated at the right of the blot.

It was reported that cAMP can activate MAPK through members of the Ras superfamily of proteins. In this mechanism, cAMP binds to the guanidine exchange factor Epac and activates Rap1, which then increases the phosphorylation of MAPK [27], [45]. Based on these evidences, we decided to study the role of the cAMP/Epac alternative pathway on the hCG leptin induction. We first determined whether the activation of the cAMP/Epac pathway induces leptin expression in placental cells. We performed cotransfection experiments with pL1951 plasmid and expression plasmids for Rap1b and Epac proteins. Results are shown in Fig. 5A. The overexpression of Rap1 alone, or both Rap1 and Epac, produced a significant induction of leptin promoter activity. In addition, we cotransfected cells with pL1951 plasmid, Epac and RapGAP, a Rap1 GTPase-activating protein which accelerates the ability of Rap1 to hydrolyze GTP into GDP [46]. The overexpression of RapGAP protein caused a significant inhibition of leptin promoter stimulation by Epac (Fig. 5B).

We next investigated the involvement of the cAMP/Epac pathway on the hCG dependent leptin induction. First, we analyzed the effects of the general cAMP analogue (Bu)_2_cAMP and the Epac selective cAMP analogue 8-(4-chloro-phenylthio)-2-Omethyladenosine-3′, 5′-cyclic monophosphate (8-CPT-2Me-cAMP, also referred in the text and figures as CPT-OMe) [47], on leptin upregulation by hCG. To this end, we treated BeWo cells with hCG, (Bu)_2_cAMP and CPT-OMe and leptin expression was assessed by Western blot. Fig. 5C shows that hCG as well as CPT-OMe produced a significant increase in leptin expression, with a 2-fold induction when compared to the control. Moreover, when hCG was combined with CPT-OMe, such stimulation was significantly higher than stimulation with hCG alone. On the other hand, treatment with hCG and (Bu)_2_cAMP inhibited leptin upregulation as previously seen. As shown in Fig. 5D, similar results were observed in placental explants. These results suggest that activation of the cAMP/Epac alternative signaling pathway, and not the PKA pathway participates in hCG upregulation of leptin. To confirm the observed results, we performed transient transfection experiments with pL1951 plasmid. As shown in Fig.5E, hCG treatment caused a significant induction in leptin promoter activity. CPT-OMe produced a 4,5-fold increase in leptin expression. When cells were treated with hCG in combination with CPT-OMe, a 3-fold induction was observed.

**Figure 6:**
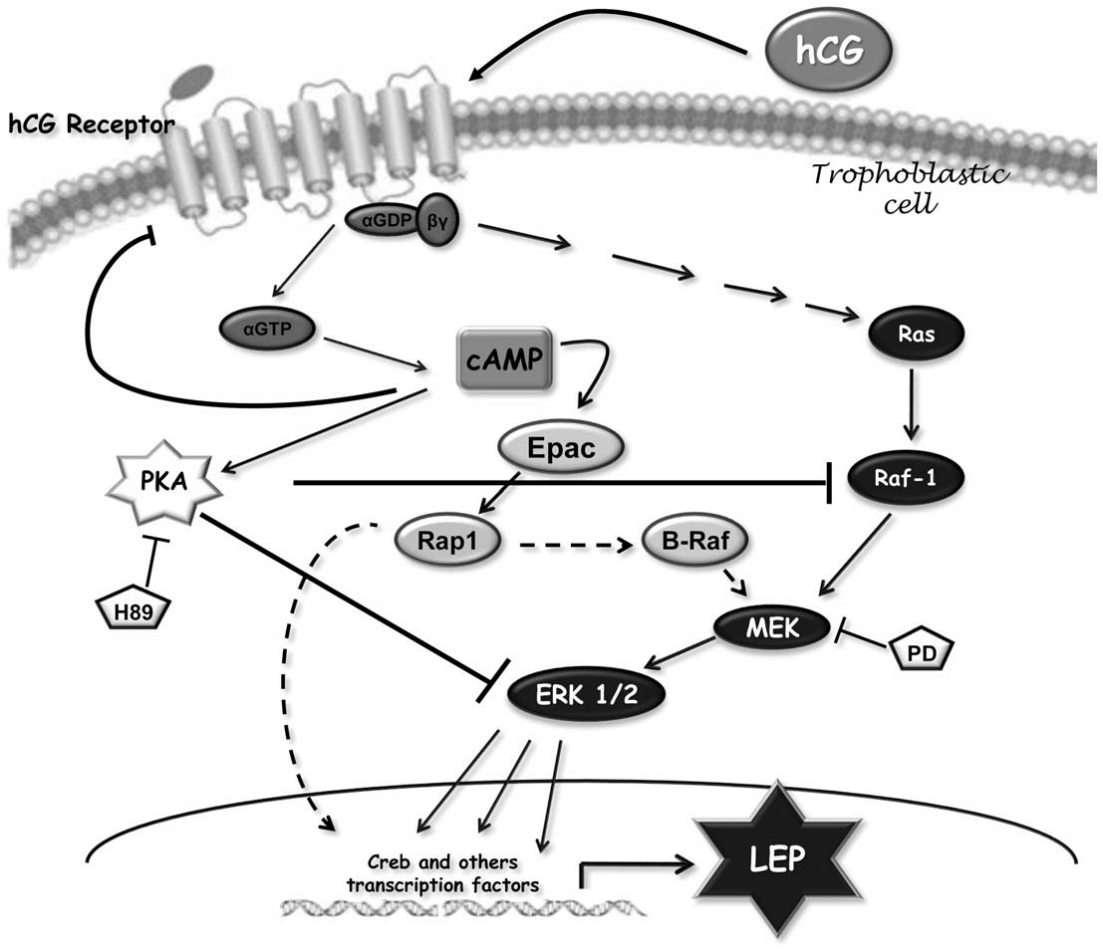
The MAPK and the alternative cAMP/Epac signaling pathways participate in leptin stimulation by hCG in placenta. Proposed model of the signaling pathways involved in hCG stimulation based on current data and its relation to leptin expression. Pointed arrow: Stimulation; Flat arrow: Inhibition. Dash arrow: possible pathways involved.

In order to study the crosstalk between the PKA, cAMP/Epac and MAPK pathways in leptin induction, BeWo cells were treated with hCG, H89 and/or CPT-OMe. Leptin expression was measured by Western blot (Fig. 5F). The cAMP analogue produced a 3,4-fold induction of leptin expression compared with control. This stimulation was enhanced when CPT-OMe was combined with hCG, reaching a 8-fold induction above control in accordance with previous results. In addition, CPT-OMe in combination with H89 caused a 5-fold increase above CPT-OMe treatment, suggesting that leptin expression involves the cAMP/Epac signaling pathway independently of PKA activation. To further investigate the involvement of this pathway in the hCG effect on leptin expression, we performed transient cotransfection experiments using the reporter pL1951 construction and the expression plasmids for the proteins Epac and Rap1. BeWo cells were treated or not with 50 IU/ml hCG. Results are shown in Fig.5G. Cotransfection with Rap1 or with both Epac and Rap1 caused a 2,33 and 3-fold induction in leptin expression, respectively. In addition, when cells were cotransfected with Epac or Rap1 and treated with hCG, we observed a significant stimulation of leptin promoter activity, reaching a 3-fold induction over control in both cases. Overexpression of both Epac and Rap1 plus hCG caused a significant 2,4-fold increase. When Epac or Rap1 were overexpressed and cells were treated with hCG, the stimulation was even higher than when treated with hCG alone. Taken together, these data suggest that hCG induction of leptin gene in placental cells is mediated not only by the MAPK signaling pathway activation but also by the alternative cAMP/Epac signaling pathway.

## Discussion

Although little is known about the exact physiological role of leptin during human pregnancy, recent observations suggest that this hormone could be a key player in the regulation of embryo implantation as well as in the maintenance of pregnancy. Leptin is synthesized in the placenta [7] and pregnancy results in elevated leptin levels [48]. Maternal plasma leptin levels decline to normal values 24 h after delivery [49]. In normal pregnancy, plasma leptin concentration was found to be in the range 7.4–19 ng/ml [7]. Many physiological roles have been suggested for leptin in human pregnancy such as regulation of placental function and development, embryo implantation and growth [14], [15], [50], [51], [52], [53], [54], [55]. Little is known about the regulation of leptin expression in placenta. In this way, it has been reported that leptin synthesis is regulated by steroid hormones [56], [57], glucocorticoids and insulin [16]. It was also demonstrated that the human leptin gene is actively engaged by hypoxia through mechanisms that are common to other hypoxia-inducible genes [58]. Moreover, we have previously shown that leptin expression in placenta is upregulated by some important pregnancy signals such as hCG, cAMP and estradiol [18], [19], [25], [59]. In particular, in the present work, we have studied the mechanisms involved in the regulation of leptin expression by hCG in BeWo and JEG-3 human choriocarcinoma cells. These cells express both leptin and its receptor [11]. They maintain many characteristics of human trophoblast cells and have been widely used to study placental cellular signaling [60], [61], [62]. Despite we used different cell lines, both JEG-3 and BeWo, are extensively used for the study of trophoblast function, and share many properties with villous trophoblasts in terms of their morphology, biochemical markers and hormone secretion [63]. The similarities between these lines and trophoblast is of particular interest in view of the difficulty involved in obtaining large amounts of freshly isolated trophoblast cells and in succeeding in transfection procedures [64]. In spite of similarities in several aspects, they differ in other characteristics, such as their proliferative activity and degree of differentiation. Thus, BeWo cells are less differentiated than JEG-3 cells, but they have higher rates of proliferation [65]. Normal trophoblastic explants from healthy donors were also studied to confirm the physiological relevance of the results. The findings in this study confirmed that hCG stimulates leptin expression in human placenta. We observed that hCG was able to increase leptin mRNA transcription not only in JEG-3 cells but also in term placental explants. HCG has many important functions during the course of pregnancy, including stimulation of progesterone production, decidualization, angiogenesis and cytotrophoblast differentiation [66], [67], [68]. HCG is already expressed in eight-cell embryos and is secreted in high local concentrations by the blastocyst entering the uterine cavity. Therefore, it is probably one of the embryonic signals involved in the embryo-maternal dialogue during the implantation window [69]. Binding of hCG to its receptor generates signal transduction through activation of the associated heterotrimeric G-proteins and, in the classical response, there is an increase in cAMP and a consequent activation of PKA [70], [71]. In this way, here we have demonstrated that hCG increases cAMP intracellular levels in placental cells. Our results are in accordance with previous reports that observed that hCG augments intracellular cAMP in placenta [72], [73]. In addition, we have found that hCG can activate CRE element activity in transient transfection experiments. The core of the CRE sequence is found in a variety of regulatory elements of genes that are activated by PKA. The main transcription factor that binds CREs is CREB, which is activated principally by phosphorylation. We have found that CREB is involved in leptin induction by hCG, suggesting that hCG stimulation of leptin could be mediated by the cAMP/PKA signaling pathway. However, there are many other signaling pathways that activate CREB. Several studies have reported that different kinases are able to phosphorylate and activate CREB [74]. In fact, we found that (Bu)_2_cAMP not only did not enhance hCG effect but even inhibited hCG-dependent leptin mRNA expression in placental cells. It was recently described that treating rats with hCG or chronically raising cAMP production, downregulate LH/CG receptor. High concentrations of hCG or cAMP negatively regulate surface receptors in ovary and testis cells, thus decreasing the abundance of all receptor transcripts [71]. The end of signaling mediated by G protein-coupled receptor due to an excess of ligand has been described both *in vivo* and *in vitro* in different cell types [75], [76]. Our results suggest that could exist alternative signaling pathways activated by hCG, that prevent the rapid desensitization of the receptor in the presence of high ligand concentrations, as occurs during implantation and pregnancy. Some authors have shown that hCG receptor is up- or down-regulated depending on the cAMP concentration used and the exposure time [40]. In this context, we demonstrated that low cAMP concentrations (<1 µM) combined with low hCG concentrations (<10 IU/ml), induce leptin placental expression. These observations strongly suggest that a rise in endogenous cAMP level interferes with hCG leptin stimulation.

It is known that the PKA pathway plays a central role in biological signaling of various hormones in the placenta, such as epinephrine, prostanoids, and hCG [77]. When we treated cells with H89, we observed that this PKA inhibitor enhanced hCG stimulation of leptin expression. Moreover, we demonstrated that this effect occurs at the transcriptional level. Based on this, we hypothesized that PKA activation by high cAMP levels might be responsible for the impairment of hCG effect. In fact, several reports have provided evidence that cAMP affects some cellular processes independently of PKA [22], [23], [24]. We have previously demonstrated that hCG stimulates leptin placental expression, at least in part, through the MAPK/ERK signaling pathway [18]. In this study, we confirmed those results, demonstrating that the inhibition of the MAPK signaling pathway (using MEK inhibitor, PD98059) completely blocks hCG induction of leptin mRNA expression. The LH/hCG receptor has been shown to mediate activation of MAPK signaling pathway [42], [43], [44], [78], [79]. In view of the obtained results, we hypothesized that cAMP inhibition of hCG effect on leptin expression could be due not only to a desensitization/down-regulation of LH/hCG receptor [71], [80] but also to an inhibition of MAPK signaling pathway generated by PKA activation. Our results demonstrated that induced ERK phosphorylation by hCG in trophoblastic cells is increased when PKA is specifically inhibited with H89. Depending on the cell type and culture conditions, the activation of PKA results in the activation or the inhibition of ERK 1/2 pathway [81], [82].

On the other hand, the activation of PKA is known to counteract the Ras/Raf-1/MEK signaling pathway [83], [84], [85], [86] that is essential to trigger ERK phosphorylation. Cyclic AMP, however, seemingly promotes ERK activity in several cell types, including 3T3- preadipocytes, ovarian granulosa cells, melanoma, pituitary cells, and neuronal cells [27], [28].

As we have demonstrated that hCG increases cAMP levels in placental cells, we studied the possibility that an alternative cAMP signaling pathway, independent of PKA, could be participating in hCG leptin up-regulation. Given that hCG induces leptin expression through the MAPK signaling pathway, we searched for the pathway linking the cAMP signal to ERK activation. Although the most important target of cAMP is PKA, recently, a cAMP-guanine nucleotide exchange factor (cAMP-GEF)/Epac, emerged as a Rap1-specific GEF [26], [27], indicating that cAMP can modulate ERKs via the Epac/Rap1/B-Raf pathway in a PKA- and Ras-independent manner [27], [28], [87], [88].

In the present work we have demonstrated that leptin expression is induced in placenta through the cAMP/Epac alternative signaling pathway by two different approaches. First the overexpression of Epac and Rap1 proteins resulted in a significant increase in leptin promoter activity. Furthermore, when trophoblastic cells were treated with CPT-OMe, a cAMP analogue that specifically activates Epac, an increase in leptin expression was observed. Our results demonstrate that cAMP stimulates placental leptin expression not only through the PKA dependent pathway, as previously demonstrated [19], but also through the cAMP/Epac alternative pathway. On the other hand, since PKA might be responsible for cAMP inhibition of hCG effect on leptin, we speculated that the cAMP/Epac alternative pathway is involved in hCG leptin induction. We found that cAMP-dependent activation of Epac1 and Rap1 but not PKA is able to induce hCG leptin expression. Indeed, stimulation of Epac with CPT-OMe caused an increase in hCG effect on leptin protein expression and leptin promoter activity. It was previously reported that besides to the inhibition of Ras-Raf-Mek cascade, PKA may negatively regulate Rap1 itself [89], thus PKA may also inhibit MAPK stimulation through the cAMP/Epac signaling pathway.

In cells expressing the Raf isoform B-Raf, cAMP is known to activate ERK via the activation of Rap1 [87], [90], [91], [92], [93]. Furthermore, it was reported that B-Raf is expressed in trophoblastic cells and ablation of B-Raf abrogated ERK phosphorylation [94]. Even though MAPK activation by Epac/Rap1/B-Raf is one possible pathway involved in leptin induction by hCG, we do not discard the involvement of other signaling pathways downstream of Rap1, independent of ERK signaling.

Although the physiological significance of Epac expression on trophoblast function remains unknown, here we have shown that Epac is an important player in the induction of leptin expression by hCG. We have presented evidence indicating that hCG stimulates leptin expression in placenta through a signal transduction pathway including Epac, Rap1, and probably B-Raf, up-stream of the MEK-ERK cascade. This pathway integrates the cAMP signal to promote leptin expression in a PKA independent manner. On the other hand, hCG could stimulate leptin through MAPK signaling pathways involving the Ras and Raf-1 proteins, in a cAMP independent manner. Therefore, the activation of the MAPK and cAMP/Epac signaling pathways underlie the stimulation of leptin expression by hCG. At the same time, hCG is capable of activating the classical cAMP/PKA pathway in placenta, but this pathway would not be involved in the stimulation of leptin by hCG. In turn, cAMP generated by the action of several effectors through the activation of PKA, would stimulate leptin. All these interrelationships are pictured in the model shown in Fig. 6.

In summary, although multiple pathways are required for leptin stimulation by cAMP, only some of them are dependent on PKA, while others, like pathways activated by hCG, would be independent of PKA. Until now, no studies have addressed the role of the cAMP/Epac in the regulation of leptin by hCG in placenta. Based on our results we can speculate that the activation of the cAMP/Epac signaling pathway would provide an alternative pathway to avoid the inhibition of leptin hCG stimulation when PKA is activated, thus ensuring leptin expression requirements during pregnancy.

The molecular actions of leptin and mechanisms coordinating its expression and activity in trophoblasts are not fully elucidated. In this work we showed a novel mechanism activated by hCG in placenta to induce leptin expression.
